# Mass spectrometric quantitation of AGEs and enzymatic crosslinks in human cancellous bone

**DOI:** 10.1038/s41598-020-75923-8

**Published:** 2020-11-02

**Authors:** Shoutaro Arakawa, Ryusuke Suzuki, Daisaburo Kurosaka, Ryo Ikeda, Hiroteru Hayashi, Tomohiro Kayama, Rei-ichi Ohno, Ryoji Nagai, Keishi Marumo, Mitsuru Saito

**Affiliations:** 1 Department of Orthopaedic Surgery, Jikei University School of Medicine, 3-25-8, Nishi-Shinbashi, Minato-ku, Tokyo, 105-8461 Japan; 2 Laboratory of Food and Regulation Biology, School of Agriculture, Tokai University, 9-1-1, Toroku, Higashi-ku, Kumamoto, 862-8652 Japan

**Keywords:** Diagnostic markers, Osteoporosis, Mass spectrometry, Pathogenesis

## Abstract

Advanced glycation end-products (AGEs) deteriorate bone strength. Among over 40 species identified in vivo, AGEs other than pentosidine were roughly estimated as total fluorescent AGEs (tfAGEs) due to technical difficulties. Using LC-QqTOF-MS, we established a system that enabled the quantitation of five AGEs (CML, CEL, MG-H1, CMA and pentosidine) as well as two mature and three immature enzymatic crosslinks. Human bone samples were collected from 149 patients who underwent total knee arthroplasty. Their clinical parameters were collected to investigate parameters that may be predictive of AGE accumulation. All the analytes were quantitated and showed significant linearity with high sensitivity and precision. The results showed that MG-H1 was the most abundant AGE, whereas pentosidine was 1/200–1/20-fold less abundant than the other four AGEs. The AGEs were significantly and strongly correlated with pentosidine, while showing moderate correlation with tfAGEs. Interestingly, multiple linear regression analysis revealed that gender contributed most to the accumulation of all the AGEs, followed by age, tartrate-resistant acid phosphatase-5b and HbA1c. Furthermore, the AGEs were negatively correlated with immature crosslinks. Mass spectrometric quantitation of AGEs and enzymatic crosslinks is crucial to a better understanding of ageing- and disease-related deterioration of bone strength.

## Introduction

**Figure 1:**
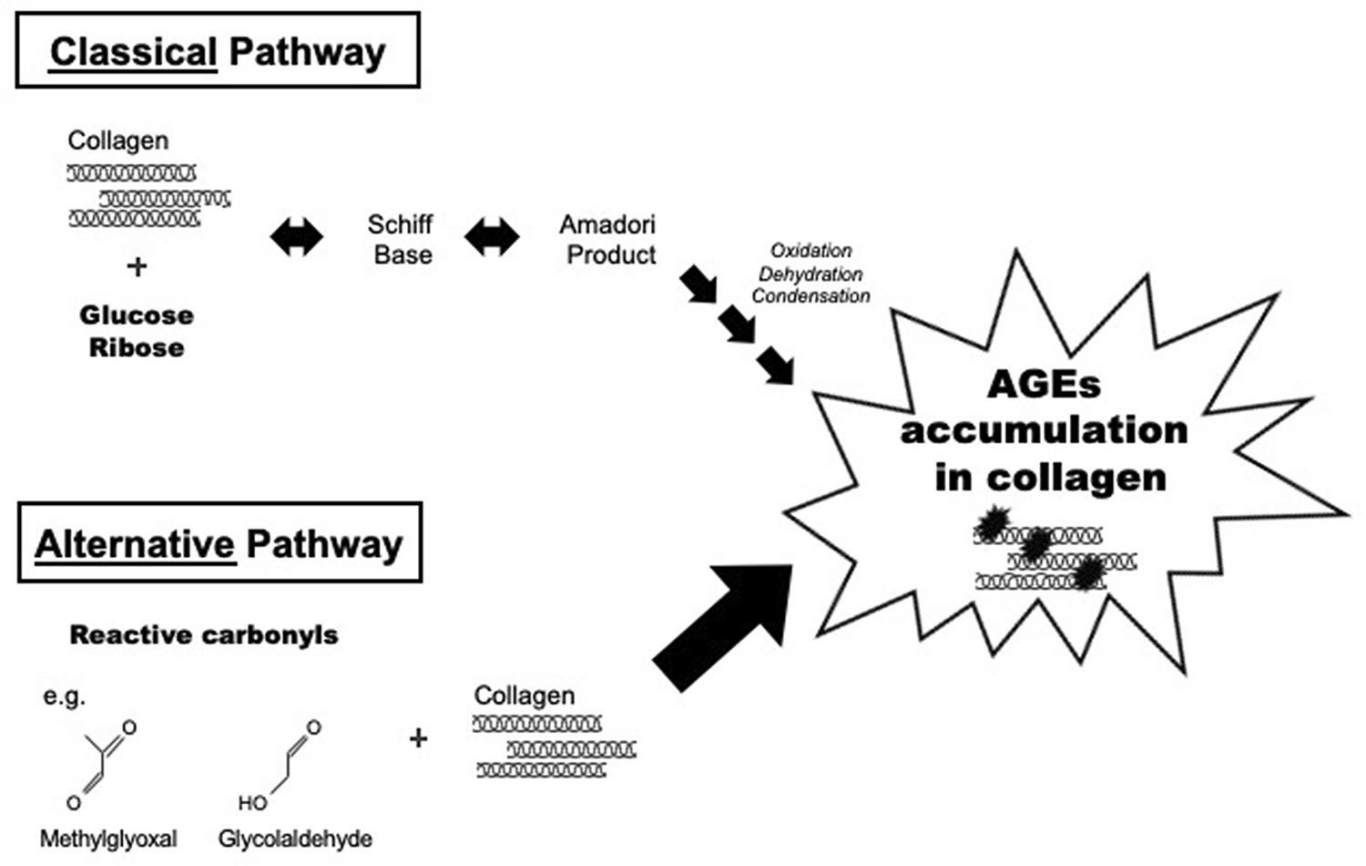
The pathways of AGEs formation.

Material property of bone is an important determinant of bone strength^1^. The nanoscale structures of bone are formed from collagen fibers surrounded and infiltrated with hydroxyapatite minerals. Collagen fibers provide the material properties such as tensile strength, ductility and toughness, while hydroxyapatite minerals are thought to contribute to stiffness. The functional properties of collagen are influenced by posttranslational modifications (PTMs). Among the modifications, the formation of enzymatic crosslinks between collagen fibrils are essential for physiological bone strength^2,3^. On the contrary, advanced glycation end-products (AGEs) are the results of non-enzymatic PTMs. AGEs are known to form via multiple pathways. In the classical pathway, reducing sugars such as glucose and ribose bind to lysine (Lys) or arginine (Arg) residues in proteins to form Schiff bases and Amadori adducts, which followed by dehydration, oxidation and condensation, eventually leading to the formation of AGEs. The alternative pathways in which reactive carbonyls (i.e. methylglyoxal, glyoxal, glycolaldehyde and glyceraldehyde) bind to Lys or Arg to form AGEs directly are postulated to be the dominant pathway compared to the classical pathway (Fig. 1)^4–6^. A series of basic and clinical trials have clarified the link between the accumulation of AGEs in bone collagen, and deterioration of bone strength. An in vitro glycation of bovine cortical bone induced pentosidine, an AGE compound, which resulted in reduced stiffness and post-yield strain as evaluated by a 3-point bending test^7^. This phenomenon was also demonstrated in human cancellous bone^8^. An in vivo study involving spontaneously diabetic rats also revealed that after the onset of diabetes, there was an increase in pentosidine accumulation in the femur with decreased bone strength despite no reduction in bone mineral density. Moreover, the link between AGEs and bone strength has been demonstrated in clinical trials. Urinary excretion of pentosidine, which is used as a surrogate marker for bone AGEs, was shown to be a predictor of vertebral fracture after adjustment for age, bone mineral density and renal function^9^. Urinary pentosidine was also shown to improve the accuracy of fracture risk classification, which used the conventional risk assessment tool (Fracture and Immobilization Score: FRISC)^10^. Increasing levels of *Nε*-(carboxymethyl)lysine (CML), another major AGE compound, in serum was also associated with hip fracture risk independent of bone mineral density^11^.

Enzymatic crosslinks have been well-characterized, and five compounds have been quantitated in bone collagen, namely, dihydroxylysinonorleucine (DHLNL), hydroxylysinonorleucine (HLNL), lysinonorleucine (LNL), pyridinoline (PYD) and deoxypyridinoline (DPD)^12–14^. DHLNL, HLNL and LNL are divalent crosslinks between collagen fibrils and are referred to as “immature” crosslinks. Immature crosslinks time-dependently turn into trivalent “mature” crosslinks, PYD and DPD. In contrast, such non-enzymatic modifications as AGEs have not been as thoroughly investigated. Although pentosidine is reported to be associated with the deterioration of bone strength, it is just one out of 40 AGEs that have been identified in vivo^2,3,12,15^. Recently, CML was detected in human bone by ELISA^16^. Other AGEs are estimated using spectrofluorometer and are referred to as total fluorescent AGEs (tfAGEs)^17^. Technological deficits continue to preclude the precise and individual quantitation of different AGEs.

**Figure 2:**
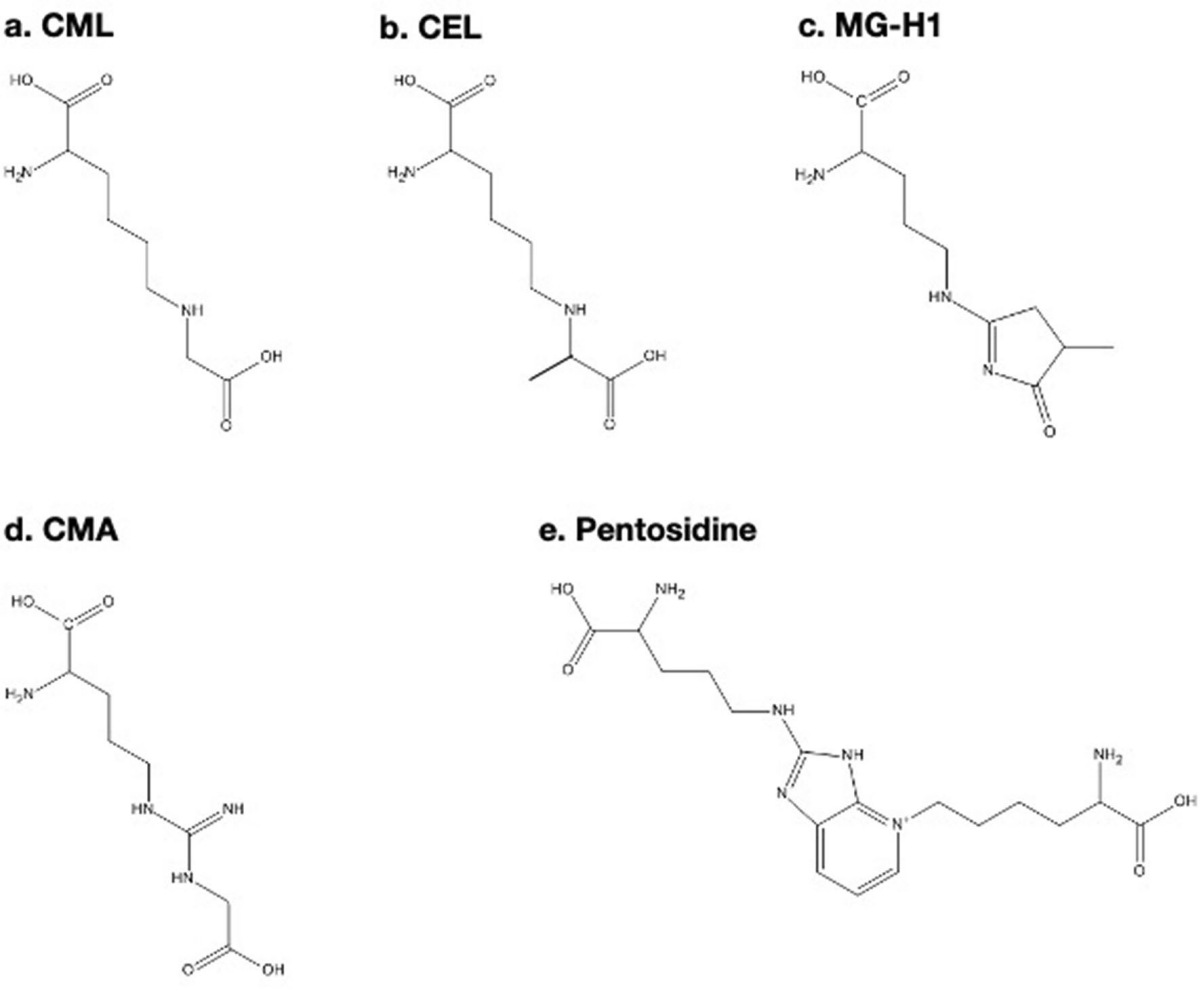
AGEs analyzed in this study. (**a**) CML, *N*^*ε*^-(carboxymethyl)lysine; (**b**) CEL, *N*^*ε*^-(carboxyethyl)lysine; (**c**) MG-H1, *N*^*δ*^-(5-hydro-5-methyl-4-imidazolon-2-yl)-ornithine 1; (**d**) CMA, *N*^*ω*^-(carboxymethyl)arginine; (**e**) pentosidine.

Advancements in analytical technologies have led to the development of mass spectrometry (MS) for the quantitation of various individual glycated adducts. Thornalley et al.^18^, Beisswenger et al.^19^, Schalkwijk et al.^20^, Morcos et al.^21^ and Monnier et al.^22^ have contributed greatly in this field. They analyzed several AGEs and early glycation products in body fluid and soft tissues such as organs and muscles, and sought their potentials as markers or even as causative substances of pathologies such as diabetic complications and chronic kidney disease. We also reported a liquid chromatography (LC)-triple quadrupole MS-based quantitation of CML^23^ and *N*^*δ*^-(5-hydro-5-methyl-4-imidazolon-2-yl)-ornithine 1 (MG-H1)^24^ in rodent bone. After technological and methodological improvements, we successfully established a system using LC-quadrupole time-of-flight (QqTOF)-MS to quantitate five AGEs and five enzymatic crosslinks in human bone collagen as depicted in Fig. 2. We also quantitated hydroxyproline (Hyp) as a surrogate of collagen content as described previously^12^. Regarding CML formation, it is generated via oxidative degradation of Amadori products, or auto-oxidation of glucose through the production of glyoxal^25^. CML also forms from glycolaldehyde, a reactive carbonyl formed from serine by the action of myeloperoxidase^26^. *N*^*ε*^-(carboxyethyl)lysine (CEL) is reported to form from the reaction between acetol with Lys^27^. CEL also forms between Lys and methylglyoxal, another reactive carbonyl generated by degradation of triosephosphate in the glycolytic system (Embden-Meyerhof pathway)^28–30^. MG-H1 forms between Arg and methylglyoxal^28^. *N*^*ω*^-(carboxymethyl)arginine (CMA) forms from Arg and glyoxal, and accumulates specifically in collagen^31^. Pentosidine derives from Lys, Arg and reducing sugars such as glucose and ribose^32,33^. Oxidation of Amadori products is also necessary for the generation of pentosidine^27,34^. An in vitro pathway has also been proposed that pentosidine forms directly from either glycolaldehyde or alternatively glyceraldehyde, a reactive carbonyl formed from oxidative degradation of glucose^35^. As described, the various patterns of AGE formation suggest that each AGE accumulation may differ in vivo, and it is of great importance to quantitate individual AGEs other than pentosidine or tfAGEs. On the other hand, quantitation of enzymatic crosslinks is also necessary as enzymatic crosslinks are determinants of bone strength with a recent report from Hudson et al. suggesting that the formation of enzymatic crosslinks are inhibited by glycation^36^.

In this study, we quantitated five AGEs and five enzymatic crosslinks in 149 human cancellous bone samples by LC-QqTOF-MS to examine the patterns of AGEs accumulation, and to investigate whether pentosidine or tfAGEs more accurately reflects the actual AGEs status in bone collagen. As the clinical manifestations of AGEs accumulation include aging^12,37^, diabetes^38^ and renal failure^39^, we also analyzed the association between AGEs and the clinical parameters such as age, gender, BMI, history of diseases, glycated hemoglobin (HbA1c) as the marker of blood glycemic status over several weeks to months^40^, tartrate-resistant acid phosphatase-5b (TRACP-5b) as the measure of bone resorption, and estimated glomerular filtration rate (eGFR).

## Results

Clinical characteristics of the patients are summarized in Table 1. There were no gender differences in age, BMI and laboratory data. The prevalence of hypertension and dyslipidemia was higher in females.

**Table 1:** Clinical characteristics of the participants.

	Total	Male	Female	*p*
	(n = 149)	(n = 31)	(n = 118)	
**Clinical**				
Age (years)	73.95 ± 8.08	74.35 ± 8.31	73.85 ± 8.05	0.762
BMI (kg/m^2^)	25.72 ± 3.61	25.34 ± 2.54	25.81 ± 3.84	0.435
**History**				
Hypertension (n, %)	78, 52.35	13, 41.94	65, 55.08	n/a
Dyslipidemia (n, %)	41, 27.52	4, 12.90	37, 31.36	n/a
**Laboratory**				
eGFR (mL/min/1.73 m^2^)	64.70 ± 13.01	66.13 ± 11.33	64.33 ± 13.44	0.454
HbA1c (%)	5.86 ± 0.41	5.76 ± 0.43	5.88 ± 0.40	0.158
TRACP-5b (mU/dL)	272.0 ± 127.2	267.3 ± 107.3	273.2 ± 132.3	0.797

Values are shown as the mean ± standard deviation, or as labeled.

*BMI* Body Mass Index, *eGFR* estimated glomerular filtration rate, *TRACP-5b* tartrate-resistant acid phosphatase-5b.

**Figure 3:**
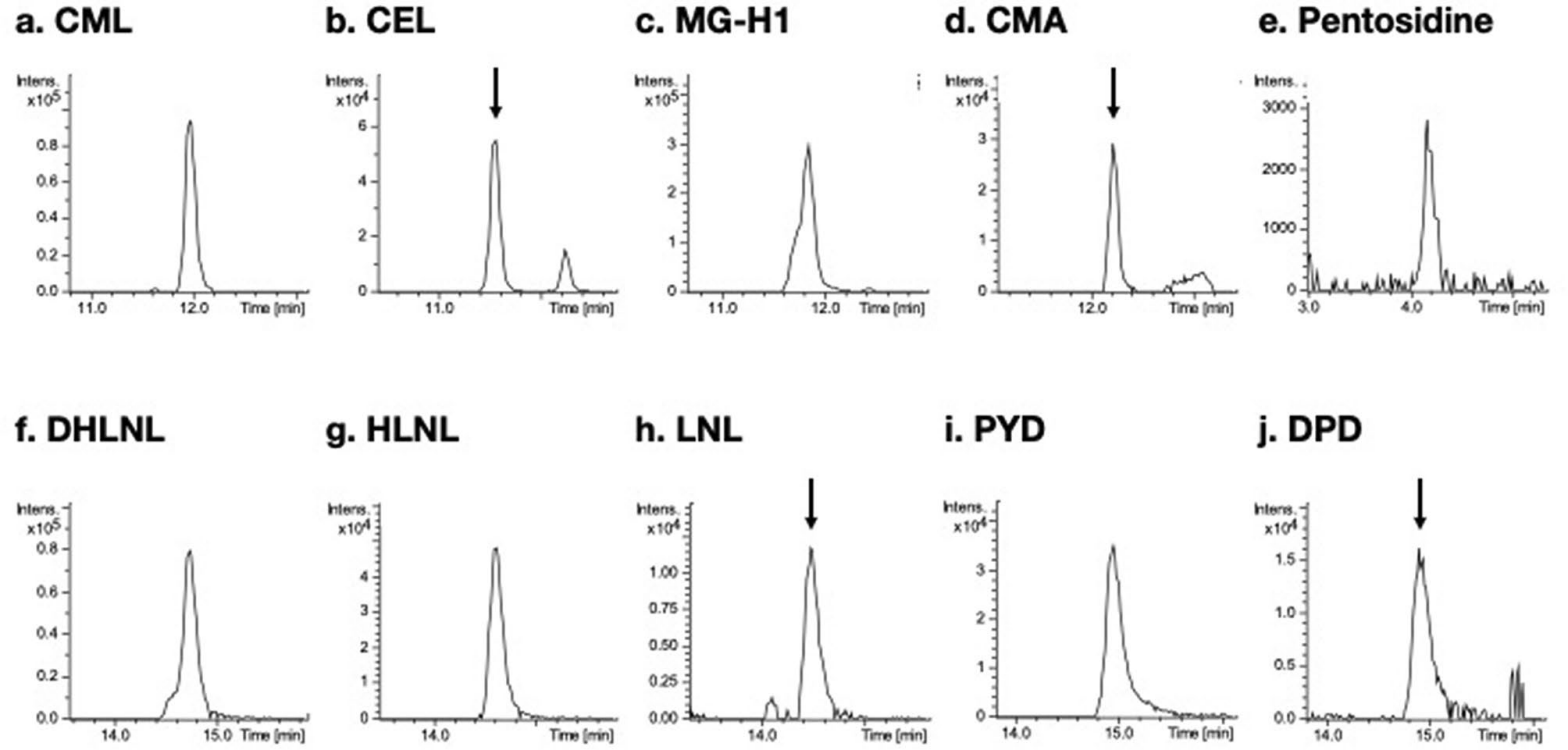
Typical extracted ion chromatographic peaks of AGEs and enzymatic crosslinks (theoretical m/z ± 0.005). (**a**) CML, *N*^*ε*^-(carboxymethyl)lysine; (**b**) CEL, *N*^*ε*^-(carboxyethyl)lysine; (**c**) MG-H1, *N*^*δ*^-(5-hydro-5-methyl-4-imidazolon-2-yl)-ornithine 1; (**d**) CMA, *N*^*ω*^-(carboxymethyl)arginine; (**e**) pentosidine; (**f**) DHLNL, dihydroxylysinonorleucine; (**g**) HLNL, hydroxylysinonorleucine; (**h**) LNL, lysinonorleucine; (**i**) PYD, pyridinoline; (**j**) DPD, deoxypyridinoline.

Typical extracted ion chromatographic peaks of the AGEs and enzymatic crosslinks in human bone hydrolysate are shown in Fig. 3. Linearity, sensitivity, precision and recovery validations are shown in Table 2. All individual AGEs demonstrated linear response when dissolved in 0.1% (v/v) formic acid with coefficients of correlation (r^2^) above 0.998 ranging from 0 to 1000 nmol/L. All individual enzymatic crosslinks also demonstrated linear response both in 0.1% (v/v) formic acid and in bone hydrolysate with r^2^ above 0.998 ranging from 0 to 250 nmol/L. Limit of detection (LOD) and limit of quantitation (LOQ) of AGEs were below 14 and 42 nmol/L, respectively. LOD and LOQ of enzymatic crosslinks were below 12 and 35 nmol/L in 0.1% formic acid, and below 16 and 48 nmol/L in bone hydrolysate. Validation of precision showed that intraday coefficients of variations (CVs) were below 5% for all analytes, whereas interday CVs were slightly greater, especially for enzymatic crosslinks in bone hydrolysate (6–12%). Recoveries of AGEs were 82–100% except for MG-H1 (60%). Recovery rates of enzymatic crosslinks were approximately 50–65%.

**Table 2:** Linearity, sensitivity, precision and recovery of the analytes.

Matrix	Analyte	Linearity		Sensitivity (nM)		Precision (%)		Recovery (%)		
		Spiked range (nM)	r^2^	LOD	LOQ	Intraday	Interday	10 (pmol)	20 (pmol)	40 (pmol)
0.1% formic acid	**Amino acid**									
	Hyp	0–250,000.00	1.000	32.43	98.27	0.50	0.63	–	–	–
	AGEs									
	CML	0–1000.00	0.998	7.74	23.45	1.72	2.19	–	–	–
	CEL	0–1000.00	1.000	13.85	41.98	1.38	1.83	–	–	–
	MG-H1	0–1000.00	1.000	6.01	18.22	1.37	1.91	–	–	–
	CMA	0–1000.00	1.000	7.69	23.30	1.95	2.42	–	–	–
	Pentosidine	0–500.00	1.000	7.95	24.09	3.18	3.22	–	–	–
	**Enzymatic crosslinks**									
	DHLNL	0–1000.00	1.000	5.26	15.95	1.65	2.53	–	–	–
	HLNL	0–1000.00	1.000	6.52	19.75	2.65	2.44	–	–	–
	LNL	0–1000.00	1.000	5.61	16.99	2.06	2.02	–	–	–
	PYD	0–1000.00	1.000	11.34	34.36	4.55	6.47	–	–	–
	DPD	0–1000.00	1.000	11.46	34.73	3.57	6.26	–	–	–
Bone hydrolysate	**Amino acid**									
	Hyp	0–250,000.00	–	10,908.52	33,056.12	2.09	4.53	8.55 ± 0.11	8.93 ± 0.03	9.39 ± 0.04
	**AGEs**									
	CML	0–250	0.997	21.20	64.23	3.37	3.46	84.64 ± 2.04	70.71 ± 0.41	71.35 ± 1.59
	CEL	0–250	0.999	13.57	41.12	4.72	3.90	92.11 ± 1.60	86.92 ± 0.93	85.93 ± 0.20
	MG-H1	0–1000	0.998	71.05	215.29	3.85	3.29	72.26 ± 0.80	64.84 ± 0.04	70.42 ± 0.46
	CMA	0–250	1.000	7.61	23.05	5.03	4.62	66.98 ± 1.05	68.13 ± 0.38	65.49 ± 0.98
	Pentosidine	0–250	0.999	10.14	30.72	3.62	4.94	100.48 ± 7.36	94.30 ± 2.53	98.08 ± 2.94
	**Enzymatic crosslinks**									
	DHLNL	0–250	1.000	9.37	28.39	2.03	12.39	58.45 ± 6.74	58.70 ± 3.59	52.89 ± 2.01
	HLNL	0–250	1.000	10.42	31.58	4.53	6.11	55.02 ± 9.15	54.76 ± 2.70	51.90 ± 2.37
	LNL	0–250	1.000	4.69	14.20	2.07	8.14	50.39 ± 1.14	52.57 ± 0.99	51.14 ± 1.01
	PYD	0–250	0.999	15.65	47.41	5.43	6.35	74.89 ± 5.51	64.07 ± 2.03	66.54 ± 2.62
	DPD	0–250	1.000	10.07	30.51	3.53	7.08	47.31 ± 3.31	53.84 ± 1.52	52.53 ± 0.64

*LOD* limit of detection, *LOQ* limit of quantitation, *Hyp* hydroxyproline, *CML N*^*ε*^-(carboxymethyl)lysine, *CEL N*^*ε*^-(carboxyethyl)lysine, *MG-H1 N*^*δ*^-(5-hydro-5-methyl-4-imidazolon-2-yl)-ornithine 1, *CMA N*^*ω*^-(carboxymethyl)arginine, *DHLNL* dihydroxylysinonorleucine, *HLNL* hydroxylysinonorleucine, *LNL* lysinonorleucine, *PYD* pyridinoline, *DPD* deoxypyridinoline.

**Figure 4:**
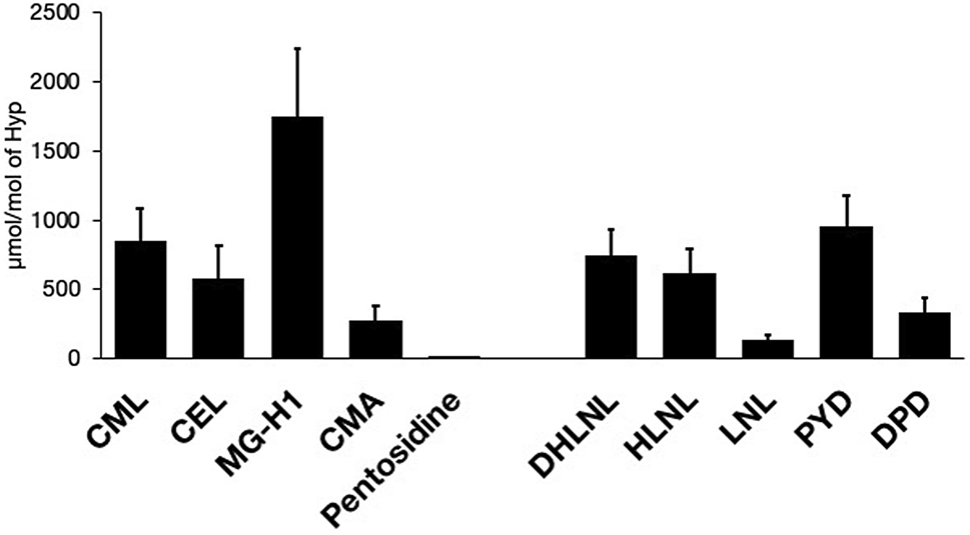
AGEs and enzymatic crosslinks in human cancellous bone from lateral tibia plateau. The filled bar and error bar reflect mean value and standard deviation. Abbreviations: Hyp, hydroxyproline; CML, *N*^*ε*^-(carboxymethyl)lysine; CEL, *N*^*ε*^-(carboxyethyl)lysine; MG-H1, *N*^*δ*^-(5-hydro-5-methyl-4-imidazolon-2-yl)-ornithine 1; CMA, *N*^*ω*^-(carboxymethyl)arginine; DHLNL, dihydroxylysinonorleucine; HLNL, hydroxylysinonorleucine; LNL, lysinonorleucine; PYD, pyridinoline; DPD, deoxypyridinoline.

MG-H1 was the most abundant AGE among the AGEs quantitated (Fig. 4). Pentosidine was 1/200–1/20-fold less abundant than the other AGEs. Analysis of gender and comorbidities revealed that male gender was associated with statistically higher AGE contents (Supplemental Table S1). The AGEs were higher in hypertensive patients but did not reach statistical significance. There were strong correlations between the AGEs; Spearman’s coefficient (r_s_) reached 0.9, except for CMA (Table 3). There were moderate correlations between the AGEs and tfAGEs with r_s_ of around 0.3.

**Table 3:** Correlation analyses between the AGEs and tfAGEs.

	CEL	MG-H1	CMA	Pentosidine	tfAGEs
	r_s_	r_s_	r_s_	r_s_	r_s_
CML	0.960***	0.933***	0.639***	0.920***	0.400***
CEL	–	0.922***	0.682***	0.886***	0.342***
MG-H1	–	–	0.639***	0.910***	0.332***
CMA	–	–	–	0.614***	0.002
Pentosidine	–	–	–	–	0.313***

*CML N*^*ε*^-(carboxymethyl)lysine, *CEL N*^*ε*^-(carboxyethyl)lysine, *MG-H1 N*^*δ*^-(5-hydro-5-methyl-4-imidazolon-2-yl)-ornithine 1, *CMA N*^*ω*^-(carboxymethyl)arginine, *tfAGEs* total fluorescent AGEs; *r*_*s*_ Spearman’s coefficient.

*p < 0.05; **p < 0.01; ***p < 0.001.

Linear regression analyses were performed to evaluate the relative contributions of clinical parameters to AGE amounts. The result showed that age and HbA1c were positively correlated, while tartrate-resistant acid phosphatase-5b (TRACP-5b)^41,42^, which is an indicator of bone resorption, was negatively correlated with the amount of each AGE (Supplemental Table S2). Subsequent multiple stepwise linear regression analysis, using the amount of each AGE as the dependent variable, with age, body mass index (BMI), estimated glomerular filtration rate (eGFR), TRACP-5b, and gender as independent variables, showed that all parameters except eGFR were independently associated with the amount of each AGE (Table 4). Gender contributed most to the accumulation of all AGEs, followed by age and TRACP-5b. HbA1c was not an independent determinant for pentosidine. Independent contributions of BMI were only significant for CML and MG-H1.

**Table 4:** Multiple stepwise linear regression analysis using each AGE content as the dependent variable, while gender, age, TRACP-5b, HbA1c, BMI and eGFR as the independent variables.

	CML	CEL	MG-H1	CMA	Pentosidine
	ß	ß	ß	ß	ß
Gender (female)	− 0.424***	− 0.414***	− 0.380***	− 0.232**	− 0.351***
Age	0.231**	0.251***	0.292***	0.194*	0.302***
TRACP-5b	− 0.285***	− 0.244***	− 0.254***	− 0.258***	− 0.278***
HbA1c	0.187**	0.212**	0.175*	0.179*	0.091
BMI	0.166*	0.095	0.158*	–	0.133
Adjusted R^2^	0.347	0.318	0.316	0.160	0.282

*CML N*^*ε*^-(carboxymethyl)lysine, *CEL N*^*ε*^-(carboxyethyl)lysine, *MG-H1 N*^*δ*^-(5-hydro-5-methyl-4-imidazolon-2-yl)-ornithine 1, *CMA N*^*ω*^-(carboxymethyl)arginine, *TRACP-5b*, tartrate-resistant acid phosphatase-5b, *ß* standardized regression coefficient, *BMI* Body Mass Index.

*p < 0.05; **p < 0.01; ***p < 0.001.

The contents of the enzymatic crosslinks are summarized in Fig. 4. PYD was the most abundant enzymatic crosslink, followed by DHLNL and HLNL. Except for HLNL, there were no differences based on gender (Supplemental Table S3). The maturation index, defined as the ratio of total mature (PYD + DPD) to total immature crosslinks (DHLNL + HLNL + LNL), was positively correlated with age (r_s_ = 0.271; p < 0.001) and negatively with TRACP-5b (r_s_ =  − 0.237; p < 0.01).

Correlations between each of the lysine-derived AGEs (i.e. CML, CEL and pentosidine), and the enzymatic cross-links were analyzed because these AGEs possibly compete with enzymatic crosslinks for formation sites^36^. The amounts of DHLNL, HLNL, and LNL were negatively correlated with CML, CEL and pentosidine, whereas no such correlation was observed for PYD and DPD (Table 5).

**Table 5:** Correlation analyses of the lys-derived AGEs (i.e. CML, CEL and pentosidine) and the enzymatic crosslinks.

	CML	CEL	Pentosidine
	r_s_	r_s_	r_s_
DHLNL	− 0.417***	− 0.471***	− 0.422***
HLNL	− 0.676***	− 0.695***	− 0.656***
LNL	− 0.285***	− 0.350***	− 0.300***
PYD	0.010	− 0.004	− 0.011
DPD	0.026	− 0.037	0.000

*CML N*^*ε*^-(carboxymethyl)lysine, *CEL N*^*ε*^-(carboxyethyl)lysine, *DHLNL* dihydroxylysinonorleucine, *HLNL* hydroxylysinonorleucine, *LNL* lysinonorleucine, *PYD* pyridinoline, *DPD* deoxypyridinoline, *r*_*s*_ Spearman’s coefficient.

*p < 0.05; **p < 0.01; ***p < 0.001.

## Discussion

In this study, the amounts of CML, CEL, MG-H1, CMA and pentosidine were quantified using LC-QqTOF-MS. Until recently, three methods were applied for the evaluation of AGEs. The first is HPLC equipped with a fluorescence detector (HPLC-FLD)^12^. This method has been reported for the quantitation of pentosidine; however, whether the quantitation of pentosidine is suitable for the elucidation of other AGEs had been unclear. The second method estimated tfAGEs^17^ using a spectrofluorometer. This method cannot detect non-fluorescent AGEs such as CML, and overestimation can occur in the presence of fluorophores other than AGEs. The third method characterizes AGEs using ELISA^16^. Although ELISA is well-characterized and conventional, a cross-reaction can occur and possibly leads to inaccurate analyses especially for AGEs. For example, 6D12, a commercially available anti-CML monoclonal antibody, can cross-react with CEL because CML and CEL are structurally similar^43^. LC–MS can potentially overcome the limitations that HPLC-FLD, spectrofluorometer and ELISA had for two reasons. First, LC–MS detects analytes in their ionized forms, thus does not need analytes to be fluorescent. Second, LC–MS can quantitate analytes with similar structures individually by the combination of retention time and mass–charge ratio (*m/z*). Although the five AGEs, whose authentic standards and isotope-labelled internal standards (ISTDs) were commercially accessible, were analyzed in this study, LC–MS will serve to the quantitation of other AGEs in the future. Among them, glucosepane is of great interest because it is thought to be a major crosslinking AGE present in various collagenous tissues^22,44^.

The developed method was validated for linearity, sensitivity, precision and recovery. All the AGEs and the enzymatic crosslinks demonstrated linear response even beyond physiological ranges. LOD and LOQ of AGEs were comparable with previous studies which utilized triple quadrupole MS^45^ and quadrupole-Orbitrap MS^46^. LOD and LOQ of enzymatic crosslinks were also satisfactory compared with the previous study using single quadrupole MS^13^. Precision validation showed that intraday CVs were below 5%, but continuous analysis lasting over three days resulted in elevated CVs. This may be due to increased solvent concentration. Therefore in this study, all samples were analyzed within 2 days. Decreased recoveries especially for enzymatic crosslinks were possibly caused by ion suppression^47^. Stable isotope dilution method and standard addition method used in this study are desirable for accurate quantitation using LC-QqTOF-MS^47,48^.

AGEs were analyzed in human bone samples in this study. It was important to take a bone sample undergoing normal remodeling because tissue remodeling is a determinant of AGE accumulation^49,50^. Cadaveric bone is ideal but is not readily available in Japan. Therefore, we took samples from the dissected tibia in total knee arthroplasty. The turnover of cancellous bone in osteoarthritic area might be disturbed, as is the case for subchondral bone^51^. We adopted the sampling method of Oren et al.^52^ and took only the cancellous bone from the central one-third and at a depth of 10 mm of the lateral tibial plateau where the superficial cartilage was macroscopically intact. Besides the sampling limitation, it was noteworthy that significant correlations were observed here between the AGE contents and age as shown in the previous studies^12,16^.

As described in the introduction, and the alternative pathways that involve reactive carbonyls (i.e. methylglyoxal, glyoxal, glycolaldehyde and glyceraldehyde) are postulated to be the dominant mechanism compared to the classical pathway (Fig. 1)^4^. To support this, the results of this study showed that MG-H1, formed from methylglyoxal and arginine, was the most abundant AGE. On the other hand, pentosidine was much less abundant. MG-H1 content in bone has never been examined previously; however, reports on serum protein^18^, muscle^18^ and Achilles tendons^53^ have shown that MG-H1 accumulation is 200–9,000 times greater than pentosidine accumulation. Methylglyoxal is 20,000 times more reactive than glucose^54^, and this high reactivity could explain the abundance of MG-H1 in various tissues. CML was the second most abundant AGE, accumulating at approximately 100 times the rate of pentosidine. Thomas et al. quantitated CML in human cortical bone using ELISA and compared it with the amount of pentosidine^16^. Although sample type (cancellous versus cortical bone) and analytical methods were different, their results were in accordance with ours in terms of the higher abundance of CML over pentosidine. For a crosslinking AGE to form, two amino acids need to be located at an adequate distance, as shown by an in silico analysis of glucosepane, one of the well-known crosslinking AGEs^22,55^. This stipulation should also apply for pentosidine and thus would have a lower chance of formation when compared with non-crosslinking AGEs such as MG-H1 and CML.

It is technically difficult to quantitate all the individual AGE compounds accumulating in bone collagen, though it is presumed that multiple AGE structures, rather than one specific structure, are involved in the deterioration of bone strength. Thus a surrogate marker such as pentosidine and tfAGEs have been widely used to estimate total AGEs accumulation in bone collagen. However, whether pentosidine or tfAGEs accurately represents other AGEs had not been thoroughly investigated. Thomas et al. found strong correlation (r^2^ = 0.87, r < 0.05) between pentosidine and CML in human cortical bone with limited sample volume (n = 5)^16^. Thus in this study, using 149 human cancellous bones, correlations between five AGEs including pentosidine and CML, and tfAGEs were analyzed. As a result, there were strong correlations between the AGEs quantitated in this study, and pentosidine was a better surrogate of other AGEs compared with tfAGEs, at least in human cancellous bone from the tibia. This result was unexpected because AGEs are formed by different pathways^4^ as described earlier, and thus prompted us to perform regression analyses to investigate potential determinants of AGEs accumulation. Surprisingly, in single and multiple regression analyses, gender was the strongest determinant of the AGEs, followed by age, TRACP-5b, HbA1c and BMI. Aging^12,38^, bone turnover^49,56^ and glycative stress^38,57^, which are closely linked, are shown to be determinants for pentosidine accumulation in bone. To the best of our knowledge, there is only one study, reporting gender difference, in which the pentosidine content of human cancellous bone from the femoral head was higher in males^58^. Barp et al*.* demonstrated that the level of oxidative stress was higher in male rats than in female rats^59^. Data have also been accumulated to show that women are less susceptible to oxidative stress^60^. It was also reported that levels of carbonyl compounds in skin were higher in male turkeys than in females, which raises the possibility that males are more prone to carbonyl stress^61^. The differences of oxidative and carbonyl stress between genders may explain the result that gender was a strong determinant of AGEs accumulation. BMI was a weak but independent determinant of CML and MG-H1. This might be explained by the obesity-related acceleration of oxidative stress seminal to the accumulation of AGEs^62^. Compared with the other AGEs, the adjusted R^2^ of CMA was less than 0.2, which implies the presence of other major determinants for CMA that were not examined in this study. It was interesting to find that, unlike other AGEs, HbA1c did not become an independent determinant of pentosidine. HbA1c is a glycated hemoglobin and used as a serological marker of glycemic status over several weeks to months^40^. Although pentosidine formation via the alternative pathway has been implicated in vitro^35^, pentosidine is still presumed to form predominantly through the classical pathway which is a longer process compared to other AGEs pathways^4^. It can be speculated that pentosidine accumulates in bone collagen in association with a longer period of hyperglycemia than required for the accumulation of other AGEs quantitated in this study. Thus HbA1c, which represents hyperglycemic status over only a few months, did not become an independent determinant of pentosidine accumulation. Overall, the strong correlations observed between individual AGE contents indicate that gender, age and bone turnover were strong independent determinants of individual AGEs, irrespective of their pathways. This may be true for the other AGEs not analyzed in this study. From the results of this study, pentosidine is shown to be a more suitable surrogate marker for AGEs accumulation in the human cancellous bone.

The amounts of PYD and DPD detected in this study were consistent with previous reports documenting human cancellous bone in which HPLC-FLD and LC–MS methods were used in conjunction with a conversion factor which assumes that collagen weighs 7.5 times the measured weight of Hyp which has a molecular weight of ~ 300,000 daltons^12,13^. The DHLNL amount of human cancellous bone observed by Gineyts et al*.*^13^ were slightly higher than ours (0.345 ± 0.063 vs 0.231 ± 0.057 mol/mol of collagen). This discrepancy might be attributable to differences in sample types (cancellous tibial bone versus cancellous bone of lumbar vertebrae) and sample extraction methods. The maturation index was positively correlated with age and negatively correlated with TRACP-5b. These results are consistent with the findings that immature crosslinks transform into mature crosslinks time-dependently^12,13^.

Interestingly, the results of this study showed that the contents of immature crosslinks were negatively correlated with the contents of Lys-derived AGEs. We previously observed that the contents of femoral pentosidine were elevated in spontaneously diabetic rats whereas immature crosslinks decreased^38^. It was postulated that a hyperglycemia-induced vitamin B6 deficiency caused the down-regulation of lysyl oxidase (LOX) and an eventual reduction in immature crosslinks. Recently, Hudson et al. reported using tail collagen from spontaneously diabetic mice that the sites where immature crosslinks began forming were almost identical to the glycation sites^36^. Thus, the formation of immature crosslinks can be blocked by glycation. In this regard, our findings support the notion of Hudson et al. In terms of abundance, the AGEs other than pentosidine were present to the same order of magnitude as enzymatic crosslinks. Crosslinking AGEs, such as pentosidine and glucosepane, crosslink randomly and can deteriorate the biochemical and mechanical properties of collagen fibrils. Non-crosslinking Lys-derived AGEs, such as CML, on the other hand, can weaken collagen fibrils by blocking the formation of immature crosslinks. With regard to non-crosslinking Arg-derived AGEs such as MG-H1, there has been no study that tried to investigate their effects on bone strength. Given their abundance, future research focusing on their effects on bone strength would be significant.

Several study limitations warrant addressing. First is regarding acid-labile AGEs. In order to determine total AGE contents in proteins using LC–MS, all peptide bonds need to be hydrolyzed. For this, acid hydrolysis in presence of 6 N HCl at 110 °C for 18–24 h is widely used^12,16,52^. However, acid-labile AGEs, such as glucosepane, break down during this process. Enzymatic hydrolysis is an alternative in this situation, although it is time consuming, expensive, and impractical for routine analysis^18^. Moreover, Antonova, Frolov et al*.*^63^ pointed out the possibility that compromised solubility of proteins could lead to insufficient enzymatic digestion and result in inaccurate quantitation of AGEs. We opted to add ISTDs before acid hydrolysis so that the losses of partially acid-labile AGEs (i.e. MG-H1 and CMA) during hydrolysis could be corrected. Secondly, the strong correlations between the AGEs in this study may be limited to cancellous bone. Thomas et al. found strong (r^2^ = 0.87, p < 0.05, n = 5) correlations between pentosidine and CML in human cortical bone^16^. Meanwhile, Karim et al. found weak correlations (r = 0.226, p < 0.05, n = 91) between pentosidine and tfAGEs in human cortical bone^17^. Factors other than bone turnover are more influential to the accumulation of AGEs because cortical bone turnover is relatively slower^64^. The quantitation of various AGEs in cortical bone is currently in progress. Thirdly, the spatial distribution of AGEs cannot be evaluated using our method of analysis. The efficient use of immunohistochemical techniques may improve the distributional evaluations of AGEs in bone tissue.

In conclusion, mass spectrometric quantitation of AGEs and enzymatic crosslinks in human cancellous bone revealed that pentosidine was 1/200–1/20-fold less abundant than the other AGEs. Unexpectedly, there were strong correlations between the individual AGEs, whereas moderate correlations were observed between the individual AGEs and tfAGEs. Pentosidine, though in itself accumulates in a relatively small quantity, may be a more suitable surrogate marker of other AGEs compared with tfAGEs. In single and multiple regression analyses, gender was the strongest determinant of the AGEs, followed by age, TRACP-5b, HbA1c and BMI. The gender difference in oxidative and carbonyl stress may explain this. In addition, CML and CEL, the non-crosslinking Lys-derived AGEs, were negatively correlated with the immature crosslinks. This result raises the possibility that non-crosslinking AGEs attribute to the deterioration of bone strength by inhibiting the formation of enzymatic crosslinks. Mass spectrometric approach has enabled a detailed analysis of individual AGEs and crosslinks, and is crucial to a better understanding of ageing- and disease-related deterioration of bone strength.

## Methods

### Chemical reagents

CML, CEL, MG-H1, [^2^H_2_]-CML, [^2^H_4_]-CEL, and [^2^H_3_]-MG-H1 were purchased from PolyPeptide Laboratories (Strasbourg, France). CMA and [^13^C_6_]-CMA were synthesized as described previously^31^. Pentosidine was purchased from Cayman Chemical Company (MI, USA).[^2^H_4_]-Pentosidine was purchased from Alsachim (Illkirch Graffenstaden, France). l-Hyp was purchased from Nacalai Tesque (Kyoto, Japan). [^2^H_3_]-l-Hyp was purchased from C D N Isotopes Inc., (Quebec, Canada). (5*S*, 5′*S*)-Dihydroxy lysinonorleucine was purchased from Toronto Research Chemicals (Toronto, Canada). Lysinonorleucine hydrochloride and (2*S*, 2′*S*, 5*S*)-5-hydroxy lysinonorleucine hydrochloride were purchased from Santa Cruz Biotechnology Inc., (CA, USA). Pyridinoline (PYD) and deoxypyridinoline (DPD) were purchased from TLC Pharmaceutical Standards (Ontario, Canada). High-performance liquid chromatography (HPLC)-grade distilled water (H_2_O), acetonitrile (MeCN) and formic acid were purchased from Nacalai Tesque (Kyoto, Japan). All other chemicals were purchased from Wako Pure Chemical Inc (Osaka, Japan).

### Sample collection

Patients recruited to this study included 149 patients with medial osteoarthritis of the knee who underwent total knee arthroplasty at Jikei University Hospital between 2014 and 2017. Patients with diabetes mellitus, rheumatic and chronic kidney disease on hemodialysis were excluded. Patients taking statins and vitamin D supplementation were also excluded. During surgery, excised tibia samples were immediately frozen and stored at − 80 °C until biochemical analysis. Blood samples were collected from patients one month prior to surgery and creatinine concentrations and HbA1c were determined subsequently. The estimated glomerular filtration rate (eGFR) was calculated using the following formula: 194 × serum creatinine^−1.094^ × age^−0.287^ (× 0.739 if female). Serum TRACP-5b was quantitated using an ELISA kit from DS Pharma Biomedical (Osaka, Japan)^65^ as an indicator of bone resorption^66^. The baseline height and weight of patients was measured for the calculation of BMI.

This study was conducted according to the Declaration of Helsinki and received approval from the Ethics Review Committee of Jikei University Hospital. Each patient provided written informed consent before participating in this study.

### Sample preparation

Cancellous bone samples were harvested from the central one-third of the lateral tibia where the superficial cartilages were macroscopically intact and at a depth of 10 mm from the cartilage surface, and pulverized using a Multi-beads Shocker (Yasui Kikai, Osaka, Japan). Subsequently, bone powders were washed with phosphate-buffered saline (0.15 mol/L NaCl in sodium phosphate buffer, pH 7.4), delipidated with chloroform/methanol (2:1, v/v) mixture during 24 h at 4 ℃ and demineralized with 0.5 mol/L EDTA in 50 mmol/L Tris buffer (pH 7.4) for 96 h at 4 ℃.

Delipidated and demineralized bone powders were reduced by sodium borohydride in sodium borate buffer (100 mmol/L of boric acid and 1 mmol/L diethylenetriamine pentaacetic acid; pH 9.1) for 4 h at ambient temperature. Isotope-labelled internal standards (ISTDs) were spiked to reduced bone powders in the following quantities: 5 nmol of [^2^H_3_]-l-Hyp, 10 pmol of [^2^H_2_]-CML, [^2^H_4_]-CEL, [^2^H_3_]-MG-H1, and [^13^C_6_]-CMA, and 20 pmol of [^2^H_4_]-pentosidine. The same quantities of ISTDs were also spiked to six different concentrations of each authentic standard to facilitate the construction of external calibration curves. Both reduced bone powders and standards were hydrolyzed in 6 N hydrochloric acid (HCl) at 110 °C for 18 h and dried using centrifugal evaporation under reduced pressure.

Dried bone hydrolysate (0.1 mg) was resuspended in H_2_O and cleaned up by a cation exchange Strata-X-C solid phase extraction (SPE) column by Phenomenex (CA, USA) to quantitate Hyp, CML, CEL, MG-H1, and CMA. The column was washed with 2% formic acid and eluted with 7% ammonia. A separate hydrolysate (5 mg) was cleaned up by a Bond Elut-Cellulose SPE from Agilent Technologies (CA, USA) to quantitate pentosidine. The column was washed with MeCN/acetic acid/H_2_O (8:1:1, v/v/v) mixture and eluted with H_2_O. The eluate was dried, re-suspended in 200 µL of 0.1% (v/v) formic acid, filtered through a 0.45 µm polytetrafluoroethylene (PTFE) membrane filter from Millex LH, Millipore (MA, USA), and 5 µL aliquots were injected into the LC–MS.

### LC–MS analysis

For analysis of Hyp, CML, CEL, MG-H1, CMA and enzymatic crosslinks, 0.1 mg of hydrolysate was loaded on a SeQuant ZIC-HILIC column (5 µm, 150 × 2.1 mm) by Merck Millipore (MA, USA), installed on a UFLC Nexera from Shimadzu (Kyoto, Japan). The separations were performed at a flow rate of 200 µL/min, at 40 °C in a linear gradient mode, with eluents A and B being water and MeCN, both containing 0.1% (v/v) formic acid. After an isocratic step at 80% eluent B for 2 min, analytes were separated in the gradient to 10% eluent B for 14 min. After a second isocratic segment (3 min at 10% eluent B) for wash, a third isocratic segment (3 min at 80% eluent B) was run for re-equilibration. For analysis of pentosidine, 5 mg of hydrolysate was loaded on a SunShell RP-AQUA column (2.6 µm, 150 × 2.1 mm) by ChromaNik Technologies (Osaka, Japan), installed on a same LC system and with the same eluents. The separation was performed at a flow rate of 200 µL/min, at 40 °C in an isocratic mode. Pentosidine was separated in an isocratic step at 0% eluent B for 4 min. After a second isocratic segment (6 min at 60% eluent B) for wash, a third isocratic segment (3 min at 0% eluent B) was run for re-equilibration.

Detection was performed using a micrOTOF-QII quadrupole time-of-flight mass spectrometer (QqTOF-MS) by Bruker Daltonics (Bremen, Germany), equipped with an electrospray ionization source. The instrument was operated in positive ion mode, using a *m/z* range of 50–1000. Capillary voltage of ion source was set to 4500 V, nebulizer gas flow was 1.6 bar, and dry gas flow was 8 L/min. Dry temperature was set at 200 °C.

Post-run calibration for each sample was performed by injecting 20 µL of mass calibrator at the beginning of each run via the 6-port diverter valve. At each calibration, the measured masses of sodium formate cluster ions were compared with theoretical ones (*m/z* 90.976645–974.813156) to achieve 5 ppm mass accuracy. Target analytes and their ISTDs are summarized in Supplemental Table S4. The extracted ion chromatograms (theoretical *m/z* ± 0.005) were constructed for target analytes and ISTDs, and peak area ratios (target/ISTD) were calculated. The amounts of target analytes in samples were determined by comparing peak area ratios with the 6-point calibration curves of the external standards spiked with ISTDs, respectively. For enzymatic crosslinks, the standard addition method was used because the ISTDs of these crosslinks were commercially unavailable^48^. A separate hydrolysate (0.05 mg) and a hydrolysate (0.05 mg) spiked with 10 pmol of LNL, 20 pmol of DHLNL, HLNL and DPD, and 30 pmol of PYD standards were analyzed. The contents of AGEs and enzymatic crosslinks were standardized to Hyp amounts to surrogate collagen amounts^12^ and expressed as µmol/mol of Hyp.

HyStar 3.2 for HPLC operation was by Shimazu (Kyoto, Japan). otofControl 4.0 for QqTOF-MS operation, Data Analysis 4.3 for data processing, and QuantAnalysis 4.3 for quantitation were by Bruker Daltonics (Bremen, Germany).

### Method validation

The LC-QqTOF-MS method was validated for linearity, sensitivity, precision recovery. Linearity of response for individual AGEs was validated in 0.1% (v/v) formic acid, while linearity for enzymatic crosslinks was validated both in 0.1% (v/v) formic acid and bone hydrolysate because enzymatic crosslinks were quantified using standard addition method. LOD and LOQ as measures of sensitivity were calculated both in 0.1% (v/v) formic acid and bone hydrolysate based on the residual standard deviation of a regression line. Results were expressed as nmol/L. To calculate intraday and interday precisions, authentic standards of all the analytes comparable to physiological concentrations spiked in 0.1% (v/v) formic acid, and a single bone hydrolysate without standard spike were measured 10 times during the same day and further five times each in two consecutive days. Results were expressed as coefficient of variation (% CV). Recoveries were calculated by spiking 10, 20 and 40 pmol of each standard into bone hydrolysate after SPE. Results were expressed as percentage. All experiments were performed in triplicate.

### Measurement of tfAGEs

Bone hydrolysate (0.5 mg) without SPE was used to measure tfAGEs using a spectrofluorometer at wavelengths of 370 nm excitation and 440 nm emission (RF-5300PC, Shimadzu, Japan) and normalized to a quinine sulfate standard^67^. TfAGEs was expressed as ng quinine/mol of Hyp.

### Statistical analysis

All data are expressed as the mean ± standard deviation (SD). Differences between groups were examined using the Student’s *t*-test. p values of < 0.05 were considered statistically significant. Spearman’s correlation coefficient (r_s_) was used to determine the strength and direction of associations between variables and simple regression analyses were performed for univariate correlations. Multiple stepwise regression analyses were performed for multivariate analyses. All statistical analyses were performed using SAS University Edition by SAS Institute Inc. (Cary, NC, USA).

## Supplementary information

Supplementary Table S1.

Supplementary Table S2.

Supplementary Table S3.

Supplementary Table S4.

## Data Availability

The datasets generated during and/or analyzed during the current study are available from the corresponding author on reasonable request.
