# Supplementary material for: Mass spectrometric quantitation of AGEs and enzymatic crosslinks in human cancellous bone
Source: Sci Rep. 2020 Nov 2;10:18774. doi: 10.1038/s41598-020-75923-8 (PMC7606603; doi:10.1038/s41598-020-75923-8)
Supplement: Supplementary file 1 — Supplementary Table S1. [file 41598_2020_75923_MOESM1_ESM.docx]

Title:

Mass spectrometric quantitation of AGEs and enzymatic crosslinks in human cancellous bone

Authors:

Shoutaro Arakawa, Ryusuke Suzuki, Daisaburo Kurosaka, Ryo Ikeda, Hiroteru Hayashi, Tomohiro Kayama, Rei-ichi Ohno, Ryoji Nagai, Keishi Marumo and Mitsuru Saito

|  | n | CML | | |  | CEL | | |  | MG-H1 | | |  | CMA | | |  | Pentosidine | | |  |
| --- | --- | --- | --- | --- | --- | --- | --- | --- | --- | --- | --- | --- | --- | --- | --- | --- | --- | --- | --- | --- | --- |
| Sex |  |  |  |  |  |  |  |  |  |  |  |  |  |  |  |  |  |  |  |  |  |
| Female | 118 | 794.6 | ± | 215.4 | ^***^ | 528.7 | ± | 212.2 | ^***^ | 1651.6 | ± | 455.3 | ^***^ | 268.8 | ± | 96.1 | ^**^ | 7.8 | ± | 2.3 | ^***^ |
| Male | 31 | 1031.9 | ± | 234.2 |  | 757.4 | ± | 269.0 |  | 2101.9 | ± | 483.4 |  | 322.0 | ± | 98.1 |  | 10.0 | ± | 2.7 |  |
| HT |  |  |  |  |  |  |  |  |  |  |  |  |  |  |  |  |  |  |  |  |  |
| – | 71 | 821.8 | ± | 240.0 |  | 553.9 | ± | 229.3 |  | 1668.6 | ± | 479.8 |  | 272.8 | ± | 95.8 |  | 7.9 | ± | 2.5 |  |
| + | 78 | 864.1 | ± | 237.9 |  | 596.7 | ± | 254.2 |  | 1815.2 | ± | 501.1 |  | 286.4 | ± | 101.2 |  | 8.6 | ± | 2.6 |  |
| DL |  |  |  |  |  |  |  |  |  |  |  |  |  |  |  |  |  |  |  |  |  |
| – | 108 | 852.1 | ± | 246.9 |  | 587.9 | ± | 247.0 |  | 1747.7 | ± | 498.5 |  | 281.3 | ± | 100.5 |  | 8.3 | ± | 2.6 |  |
| + | 41 | 822.4 | ± | 218.3 |  | 545.7 | ± | 231.6 |  | 1739.1 | ± | 491.3 |  | 276.3 | ± | 94.4 |  | 8.0 | ± | 2.3 |  |

**Supplemental Table S1. Comparative analysis of gender and comorbidities.**Values are shown as the mean ± standard deviation. Units are µmol/mol of Hyp.

Abbreviations: CML, *N^ε^*-(carboxymethyl)lysine; CEL, *N^ε^*-(carboxyethyl)lysine; MG-H1, *N^δ^*-(5-hydro-5-methyl-4-imidazolon-2-yl)-ornithine 1; CMA, *N^ω^*-(carboxymethyl)arginine; HT, hypertension; DL, dyslipidemia.
^*^ p<0.05; ^**^ p<0.01; ^***^ p<0.001.
