# Supplementary material for: Mass spectrometric quantitation of AGEs and enzymatic crosslinks in human cancellous bone
Source: Sci Rep. 2020 Nov 2;10:18774. doi: 10.1038/s41598-020-75923-8 (PMC7606603; doi:10.1038/s41598-020-75923-8)
Supplement: Supplementary file 2 — Supplementary Table S2. [file 41598_2020_75923_MOESM2_ESM.docx]

Title:

Mass spectrometric quantitation of AGEs and enzymatic crosslinks in human cancellous bone

Authors:

Shoutaro Arakawa, Ryusuke Suzuki, Daisaburo Kurosaka, Ryo Ikeda, Hiroteru Hayashi, Tomohiro Kayama, Rei-ichi Ohno, Ryoji Nagai, Keishi Marumo and Mitsuru Saito

|  | CML | | CEL | | MG-H1 | | CMA | | Pentosidine | |
| --- | --- | --- | --- | --- | --- | --- | --- | --- | --- | --- |
|  | r_s_ | | r_s_ | | r_s_ | | r_s_ | | r_s_ | |
| Age | 0.153 |  | 0.170 | ^*^ | 0.223 | ^**^ | 0.176 | ^*^ | 0.251 | ^**^ |
| BMI | 0.211 |  | 0.149 |  | 0.178 |  | – 0.032 |  | 0.189 |  |
| eGFR | – 0.042 |  | – 0.048 |  | – 0.077 |  | – 0.095 |  | – 0.112 |  |
| HbA1c | 0.271 | ^***^ | 0.256 | ^**^ | 0.232 | ^**^ | 0.169 | ^*^ | 0.191 |  |
| TRACP-5b | – 0.372 | ^***^ | – 0.346 | ^***^ | – 0.340 | ^***^ | – 0.295 | ^***^ | – 0.340 | ^***^ |

**Supplemental Table S2. Linear regression analysis of the relations between the AGEs and clinical parameters.**Abbreviations: CML, *N^ε^*-(carboxymethyl)lysine; CEL, *N^ε^*-(carboxyethyl)lysine; MG-H1, *N^δ^*-(5-hydro-5-methyl-4-imidazolon-2-yl)-ornithine 1; CMA, *N^ω^*-(carboxymethyl)arginine; BMI, body mass index; eGFR, estimated glomerular filtration rate; TRACP-5b, tartrate-resistant acid phosphatase-5b; r_s_, Spearman’s coefficient.
^*^ p<0.05; ^**^ p<0.01; ^***^ p<0.001.
