# Supplementary material for: Mass spectrometric quantitation of AGEs and enzymatic crosslinks in human cancellous bone
Source: Sci Rep. 2020 Nov 2;10:18774. doi: 10.1038/s41598-020-75923-8 (PMC7606603; doi:10.1038/s41598-020-75923-8)
Supplement: Supplementary file 3 — Supplementary Table S3. [file 41598_2020_75923_MOESM3_ESM.docx]

Title:

Mass spectrometric quantitation of AGEs and enzymatic crosslinks in human cancellous bone

Authors:

Shoutaro Arakawa, Ryusuke Suzuki, Daisaburo Kurosaka, Ryo Ikeda, Hiroteru Hayashi, Tomohiro Kayama, Rei-ichi Ohno, Nagai, Keishi Marumo and Mitsuru Saito

|  | n | DHLNL | | |  | HLNL | | |  | LNL | | |  | PYD | | |  | DPD | | |  |
| --- | --- | --- | --- | --- | --- | --- | --- | --- | --- | --- | --- | --- | --- | --- | --- | --- | --- | --- | --- | --- | --- |
| Sex |  |  |  |  |  |  |  |  |  |  |  |  |  |  |  |  |  |  |  |  |  |
| Female | 118 | 753.6 | ± | 182.7 |  | 628.9 | ± | 166.6 | ^*^ | 134.2 | ± | 33.9 |  | 965.0 | ± | 218.5 |  | 332.8 | ± | 120.1 |  |
| Male | 31 | 715.7 | ± | 195.1 |  | 556.1 | ± | 201.6 |  | 134.4 | ± | 38.6 |  | 923.0 | ± | 217.2 |  | 318.4 | ± | 88.4 |  |
| HT |  |  |  |  |  |  |  |  |  |  |  |  |  |  |  |  |  |  |  |  |  |
| – | 71 | 752.7 | ± | 210.9 |  | 616.7 | ± | 182.2 |  | 134.6 | ± | 31.5 |  | 910.9 | ± | 203.1 | ^*^ | 316.0 | ± | 91.6 |  |
| + | 78 | 739.3 | ± | 159.6 |  | 611.1 | ± | 171.8 |  | 134.0 | ± | 37.8 |  | 997.5 | ± | 224.5 |  | 342.3 | ± | 130.2 |  |
| DL |  |  |  |  |  |  |  |  |  |  |  |  |  |  |  |  |  |  |  |  |  |
| – | 108 | 760.0 | ± | 201.9 |  | 617.7 | ± | 190.0 |  | 135.7 | ± | 36.7 |  | 961.6 | ± | 229.1 |  | 332.1 | ± | 116.2 |  |
| + | 41 | 708.0 | ± | 126.4 |  | 603.4 | ± | 135.0 |  | 130.5 | ± | 29.4 |  | 942.2 | ± | 188.3 |  | 323.5 | ± | 108.6 |  |

**Supplemental Table S3. Comparative analysis of gender and comorbidities.**Values are shown as the mean ± standard deviation. Units are µmol/mol of Hyp.

Abbreviations: DHLNL, dihydroxylysinonorleucine; HLNL, hydroxylysinonorleucine; LNL, lysinonorleucine; PYD, pyridinoline; DPD, deoxypyridinoline; HT, hypertension; DL, dyslipidemia.
^*^ p<0.05; ^**^ p<0.01; ^***^ p<0.001.
