# Supplementary material for: Mass spectrometric quantitation of AGEs and enzymatic crosslinks in human cancellous bone
Source: Sci Rep. 2020 Nov 2;10:18774. doi: 10.1038/s41598-020-75923-8 (PMC7606603; doi:10.1038/s41598-020-75923-8)
Supplement: Supplementary file 4 — Supplementary Table S4. [file 41598_2020_75923_MOESM4_ESM.docx]

Title:

Mass spectrometric quantitation of AGEs and enzymatic crosslinks in human cancellous bone

Authors:

Shoutaro Arakawa, Ryusuke Suzuki, Daisaburo Kurosaka, Ryo Ikeda, Hiroteru Hayashi, Tomohiro Kayama, Rei-ichi Ohno, Ryoji Nagai, Keishi Marumo and Mitsuru Saito

| Analyte | Compositional formula | Theoretical m/z | Isotope-labelled internal standard | Theoretical m/z |
| --- | --- | --- | --- | --- |
| Amino acid |  |  |  |  |
| Hyp | C_5_H_9_NO_3_ | 132.0655 (M + H^+^) | [^2^H_3_]Hyp | 135.0844 (M + H^+^) |
|  |  |  |  |  |
| AGEs |  |  |  |  |
| CML | C_8_H_16_N_2_O_4_ | 205.1183 (M + H^+^) | [^2^H_2_]CML | 207.1308 (M + H^+^) |
| CEL | C_9_H_18_N_2_O_4_ | 219.1339 (M + H^+^) | [^2^H_4_]CEL | 223.1590 (M + H^+^) |
| MG-H1 | C_9_H_16_N_4_O_3_ | 229.1295 (M + H^+^) | [^2^H_3_]MG-H1 | 232.1483 (M + H^+^) |
| CMA | C_8_H_16_N_4_O_4_ | 233.1244 (M + H^+^) | [^13^C_6_]CMA | 239.1446 (M + H^+^) |
| Pentosidine | C_17_H_26_N_6_O_4_ | 190.1080 (M + 2H^+^) | [^2^H_4_]Pentosidine | 192.1206 (M + 2H^+^) |
|  |  |  |  |  |
| Enzymatic crosslinks | |  |  |  |
| DHLNL | C_12_H_26_N_3_O_6_ | 308.1816 (M + H^+^) | – | – |
| HLNL | C_12_H_26_N_3_O_5_ | 292.1867 (M + H^+^) | – | – |
| LNL | C_12_H_26_N_3_O_4_ | 276.1918 (M + H^+^) | – | – |
| PYD | C_18_H_30_N_4_O_8_ | 215.1026 (M + 2H^+^) | – | – |
| DPD | C_18_H_30_N_4_O_7_ | 207.1052 (M + 2H^+^) | – | – |

**Supplemental Table S4. Compositional formulas and theoretical mass-charge ratios (m/z) of the analytes and their isotope-labelled internal standards.**

Abbreviations: CML, *N^ε^*-(carboxymethyl)lysine; CEL, *N^ε^*-(carboxyethyl)lysine; MG-H1, *N^δ^*-(5-hydro-5-methyl-4-imidazolon-2-yl)-ornithine 1; CMA, *N^ω^*-(carboxymethyl)arginine; DHLNL, dihydroxylysinonorleucine; HLNL, hydroxylysinonorleucine; LNL, lysinonorleucine; PYD, pyridinoline; DPD, deoxypyridinoline.
